# Pacific oral health: a scoping review

**DOI:** 10.3389/froh.2025.1474623

**Published:** 2025-03-25

**Authors:** Naailah Zahraa Hanif, Zac Morse, Jonathan Broadbent, Anumala Ram

**Affiliations:** ^1^ Faculty of Health and Environmental Sciences, Auckland University of Technology, Auckland, New Zealand; ^2^ American University of Iraq—Baghdad, Baghdad, Iraq; ^3^ Faculty of Dentistry, Sir John Walsh Research Institute, University of Otago, Dunedin, New Zealand; ^4^ College of Medicine, Nursing and Health Sciences, Fiji National University, Suva, Fiji

**Keywords:** dental caries, epidemiology, health disparities, oral health conditions, Pasifika, periodontal disease, tooth loss

## Abstract

**Introduction:**

A growing body of literature reports on the oral health of Pacific peoples but a synthesis of the existing knowledge on Pacific oral health epidemiology is absent. This scoping review aims to summarise the evidence on Pacific oral health epidemiology. The findings of this review may help identify knowledge gaps and issues requiring health policy prioritisation.

**Methods:**

The review followed the PRISMA-ScR guidelines for scoping reviews and included reports published prior to July 2023 on Pacific oral health, regardless of design. Searches were conducted across four databases, and the grey literature.

**Results:**

An analysis of 95 sources, primarily from 2000 to 2023 and predominantly New Zealand-based, found that a high proportion of Pacific peoples (including children) were affected by poor oral health and challenges in accessing dental care services. Numerous studies have reported oral health disparities, with poorer oral health among Pacific peoples than other population groups. Epidemiological and health services data from Pacific Island nations show a high prevalence of dental conditions, along with limited healthcare resources and workforce shortages. Studies on the broader social determinants shaping these issues and health promotion strategies to address them were limited.

**Conclusion:**

This review revealed significant unmet oral health needs, ethnic disparities in oral health, and barriers preventing care in Pacific populations. The findings emphasise the need for more research to address these gaps to help develop effective, culturally-informed oral health strategies for Pacific communities.

## 1 Introduction

Pacific Island Countries and Territories (PICTs) comprise approximately 25,000 islands and are home to more than 3.2 million people (1, 2). These islands have diverse histories, cultures, economies, and political systems (2). The South Pacific Ocean is sparsely populated and home to a combination of Melanesians, Micronesians, Polynesians, and migrants from Asia and Europe. Indigenous island inhabitants and diaspora populations are collectively referred to as “Pacific people”.

Pacific people disproportionately experience poor oral health relative to other people in the Pacific who are of European or other non-Pacific ethnic origin (3–6). Pacific People face a triple disease burden of injuries, communicable diseases, and a rapidly rising epidemic of non-communicable diseases (2), but there is limited research on the oral health of Pacific peoples (7–9).

A scoping review involves a broad investigation of the availability of the literature on a particular issue. To support policy and practice, a scoping review of Pacific oral health helps summarise the existing knowledge base. By understanding the historical and current oral health of the Pacific, dental professionals, health policymakers, researchers, and other stakeholders can identify priority areas to improve the design of strategies and delivery of oral health care for Pacific people. Thus, this review aims to report on the nature and extent of available evidence on Pacific oral health and briefly synthesise this knowledge to inform health policy and future research.

## 2 Methods

### 2.1 Protocol and strategy

This scoping review was guided by the Preferred Reporting Items for Systematic Reviews and Meta-Analyses (PRISMA-ScR) Extension for Scoping Reviews checklist (10, 11). A preliminary search was undertaken using the EBSCO health database to identify keywords to support the search strategy. The finalised specific search terms for each search are listed in Table 1. Four health databases (Cumulative Index to Nursing and Allied Health Literature Source, Dentistry & Oral Sciences Source, SPORTDiscus and MEDLINE) were used to find relevant published literature. Additional searches were conducted via Google Scholar and Google Search to identify further relevant “grey” literature. For the latter, the first 100 sources were considered. The search included information up to July 2023.

**Table 1:** Search terms and strategy.

Search	Search terms
EBSCO health databases 1. Dentistry & oral sciences source 2. MEDLINE 3. CINAHL source 4. SPORTDiscus with full text	[Abstract] “Oral Health” OR “Oral Care” OR Dental OR “Oral Hygiene” OR periodont* OR caries OR “Oral Cavity” AND [Abstract] Pacific* OR Pasifika OR Pasefika No date restrictions No language restrictions
Google scholar	(Pacific OR Pasifika OR Pasefika OR Fiji* OR Samoa* OR Tonga*) AND (“oral health” OR “oral hygiene” OR “oral cavity” OR periodont* OR dental OR Caries)
Google search 1	“Pacific Oral Health”
Google search 2	(Pacific OR Pasifika OR Pasefika OR Fiji OR Samoa OR Tonga) AND (oral health OR oral hygiene OR oral cavity OR periodontal OR periodontitis OR dental OR Caries)

### 2.2 Eligibility criteria

Articles or studies that reported on Pacific people's oral health, regardless of the design, location, or publication date, were included. However, studies combining or categorising Pacific people's data with those of other ethnic groups were excluded. Editorials, letters, and other opinion commentary were also excluded. The search was conducted in English, but no restrictions were placed on the publication language.

### 2.3 Screening and selection process

The titles and abstracts of all sources identified from the searches were retrieved and screened by the first author. For sources with unclear abstracts, full-text documents were retrieved and screened. All sources were exported into the reference software Endnote X9 (The EndNote Team. EndNote. EndNote X9 ed. Philadelphia, PA: Clarivate. 2013) and categorised into three groups: “inclusions”, “exclusions” and “maybe”. The first and second authors then independently screened the categories. To resolve any disagreements on inclusion and exclusion, the authors discussed these differences until a consensus was reached.

### 2.4 Data extraction and analysis

The included sources underwent full-text review, with the relevant details of each source recorded by the first author using a Microsoft Excel spreadsheet. Before its development, the data charting form underwent refinement through a data calibration exercise. In this exercise, three authors independently recorded data from a subset of the five most recent sources. The full data extraction was performed by the first author, with oversight from the second author, to ensure consistency and accuracy. The data recorded included the year of publication, author(s), first author's location, title, keywords, journal or publisher name, aim(s) or objective(s), type of study, location reported, Pacific population, number of participants, key findings, and future implications for research or practice. Reasons for disagreements regarding study selection and data extraction were recorded and resolved by consensus and discussion between the first three authors.

Extracted information was analysed to characterise the studies and synthesise recurring themes related to Pacific oral health.

The keywords for each source were retrieved from EndNote X9. The Journal subject term(s) for each source were retrieved from the National Centre for Biotechnology Information website (12). The key findings were summarised in the data charting form (Supplementary File A).

## 3 Results

### 3.1 Literature search and selection

**Figure 1:**
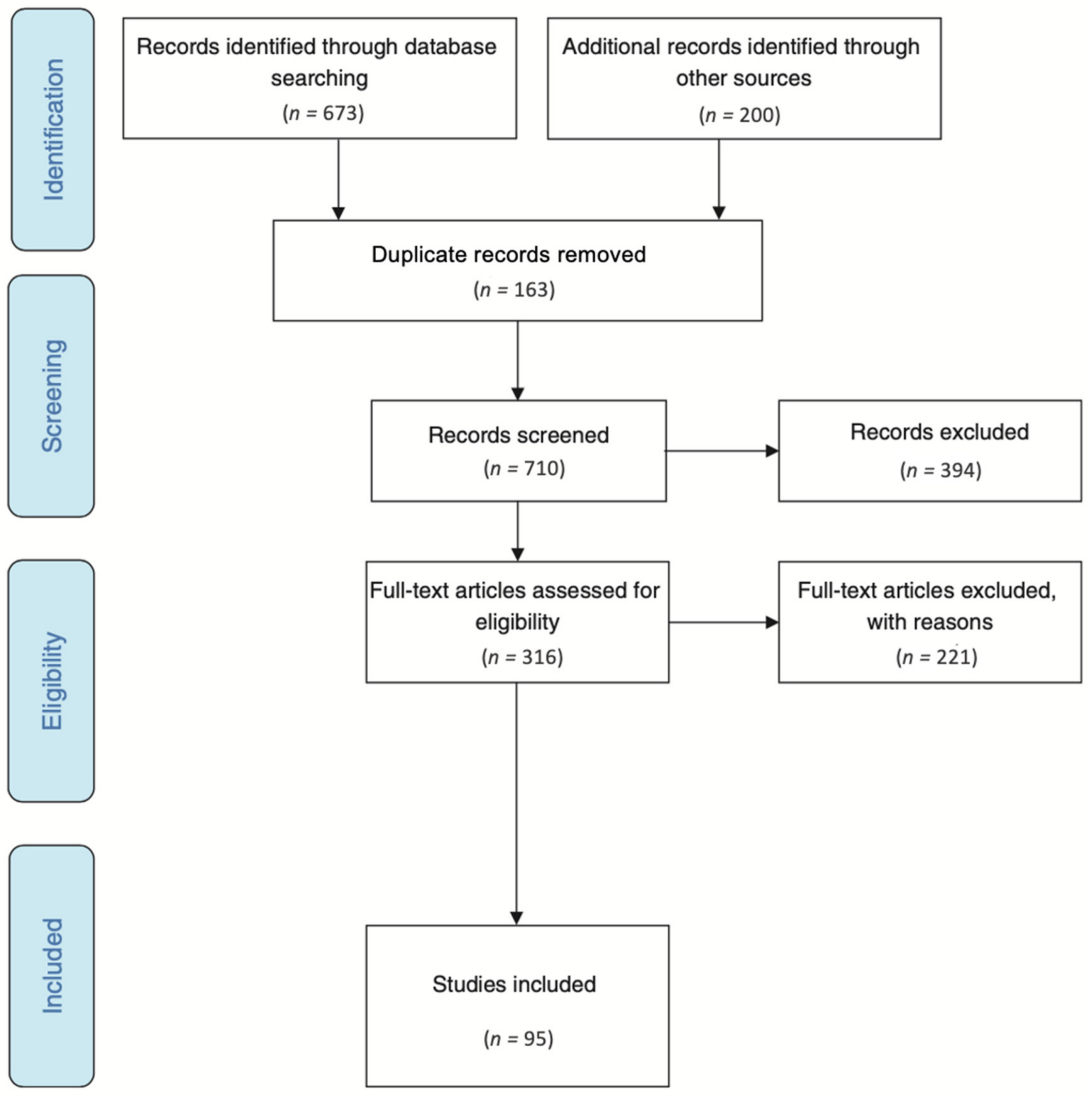
PRISMA flow chart results.

The PRISMA flow chart (13) in Figure 1 outlines the identification, screening, and inclusion processes. A total of 673 published articles were retrieved from the EBSCO health databases, including Dentistry & Oral Sciences Source (*n* = 287), MEDLINE (*n* = 254), CINAHL Complete (*n* = 124) and SPORTDiscus (*n* = 8). Additionally, the first 100 results from Google and Google Scholar were also identified, resulting in a combined total of 873 items. After removing 163 duplicates, 710 sources underwent screening. Of these, 394 were excluded because they did not report Pacific oral health. A further 221 were excluded as they were reviews with data from sources already included or reported on combined ethnicities, leaving 95 studies meeting the inclusion criteria for analysis.

### 3.2 Study characteristics

The included studies (*n* = 95) were published from 1956 to 2023, with an increasing frequency over time (Table 2). Over 80% of the included sources were published from 2000 to 2023 (*n* = 79, 83.2%), and just under 15% were published from 1956 to 1999 (*n* = 14), while the year of publication was not available for the remaining two sources (*n* = 2).

**Table 2:** Number of publications by year from 1956 to 2023.^a^

Year	Number of publications	Percentage (%)
1955–1959	1	1.1%
1960–1964	0	0.0%
1965–1969	0	0.0%
1970–1974	1	1.1%
1975–1979	3	3.2%
1980–1984	2	2.2%
1985–1989	0	0.0%
1990–1994	3	3.2%
1995–1999	4	4.3%
2000–2004	15	16.1%
2005–2009	13	14.0%
2010–2014	13	14.0%
2015–2019	18	19.4%
2020–2023	20	21.5%
Total	93^a^	100.0%

^a^ Excludes two publications for which publication date was not stated.

### 3.3 Populations studied

Over half of the studies were conducted in New Zealand (*n* = 58), while one in five were reported in different Pacific Island countries *(n* = 21*)*. Of the studies conducted in Pacific nations, most were from Fiji (*n* = 7), followed by the Marshall Islands (*n* = 3), New Caledonia (*n* = 2) and Tonga (*n* = 2). One source each was reported in Cook Islands, Guam, Hawaii, Palau, Papua New Guinea, Samoa, Solomon Islands, and Tuvalu. Two sources were reported from the United States. The remaining 14 sources were reported across more than one country, with nine of these covering multiple PICTs.

Although all studies reported on Pacific oral health, most (*n* = 68) did not clearly specify which specific Pacific population(s) were studied. A fraction of the studies focused exclusively on the oral health of Pacific peoples (*n* = 27), while the remainder of studies included categories of other ethnic groups, including Pacific peoples. A large proportion of studies reported on New Zealand Pacific people (*n* = 58*)*. Of the New Zealand-based studies, the majority focused on child or adolescent oral health (*n* = 33), while less reported on mixed age groups (*n* = 19) and New Zealand Pacific adults (*n* = 6). A smaller proportion of studies reported on various subgroups of Pacific ethnicities, including Pacific peoples from Palau, Solomon Islands, Marshall Islands, New Caledonia, Tonga, Tuvalu, Fiji, Samoa, Hawaii, Cook Islands, and Papua New Guinea.

Almost half of the sources included in the review reported on children or adolescents aged anywhere from 0 to 17 years of age (*n* *=* *45*), while one in five reported on adults (*n* *=* *18*) and the remainder reported on multiple age groups (*n* *=* *32*). Studies that did not specify the age group of participants were also included in the all-age-group category for this review.

### 3.4 Journal and publishers

The highest number of included sources was published in the New Zealand Dental Journal (*n* *=* *14)*, followed by the Pacific Health Dialog (*n* = 11). Thirteen sources were published in a journal dedicated to dental health, five sources were published by a Ministry of Health, and two sources were reports published by universities. Most of the remaining journals were health- and science-based.

### 3.5 Investigators

The majority of the first-named authors were New Zealand-based (*n* = 56), followed by authors based in Australia (*n* = 10), Fiji (*n* = 8), and the United States (*n* *=* *5*). The remaining first-named authors were based in France (*n* = 1), Hawaii (*n* = 2), Japan (*n* = 2), the Marshall Islands (*n* = 3), the Netherlands (*n* = 1), New Caledonia (*n* = 2), Switzerland (*n* = 2), Thailand (*n* = 1), the United Kingdom (*n* = 1) and Zimbabwe (*n* = 1).

### 3.6 Search and study types

The sources were most frequently identified through database searches (*n* = 71). A smaller proportion of studies were identified through Google searches (*n* = 17) and Google Scholar (*n* = 7).

Most of the studies were cross-sectional (*n* = 33) or exclusively secondary data analyses or presentations (*n* = 32). Fewer studies were reviews (*n* = 14) or cohort studies (*n* = 9*)*. The remaining studies were less than 3%, case study (*n* = 1), discourse analysis (*n* = 1), field trial (*n* = 1), methodological framework (*n* = 1), mixed methods (*n* = 2), national statistics (*n* = 1), and nonrandomised control trial (*n* = 1).

### 3.7 Key themes

**Figure 2:**
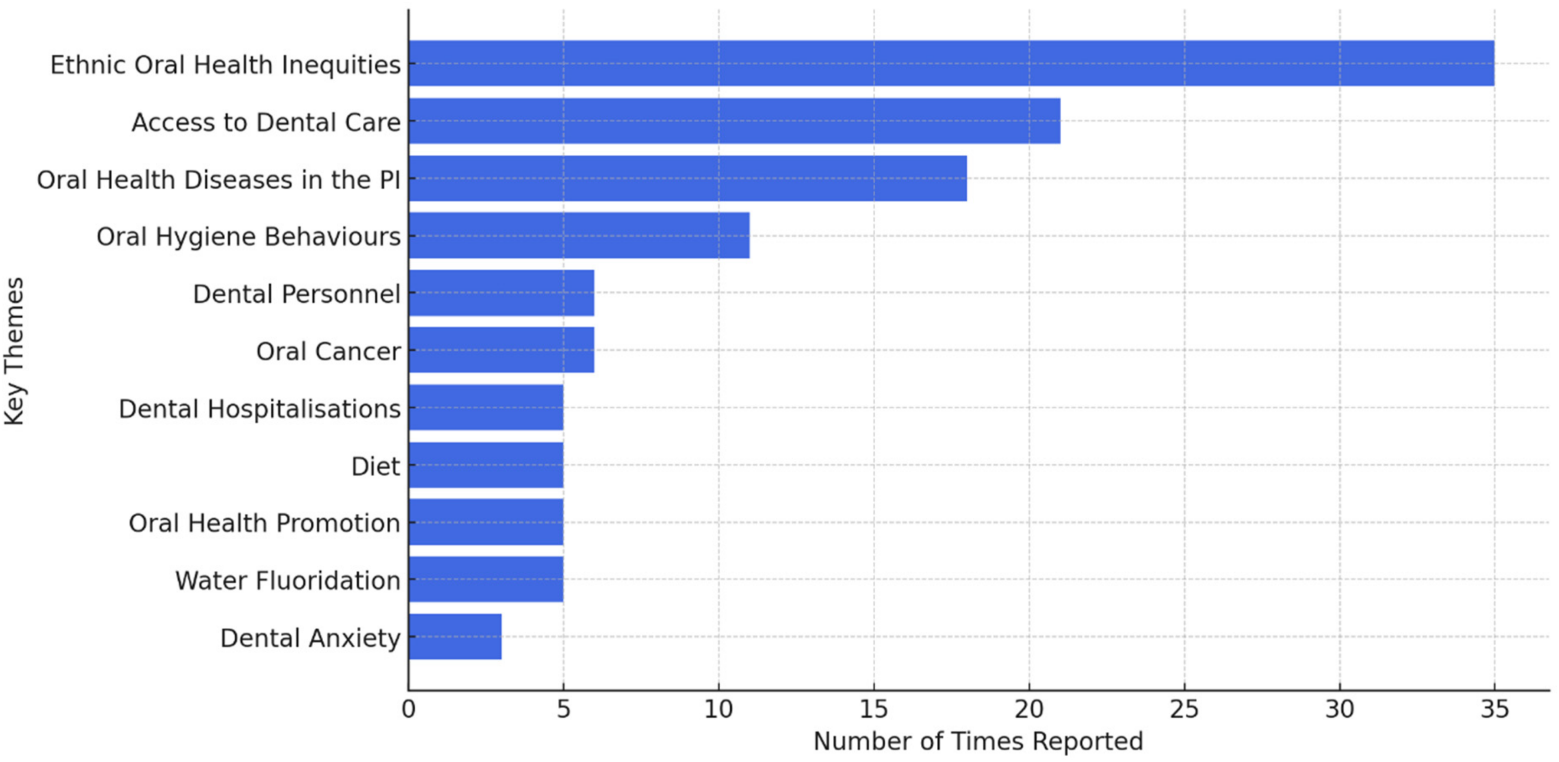
Key themes by the number of times reported.

In the analysis of key themes, some sources covered multiple themes. Specifically, 26 sources (27.4%) addressed more than one key theme, resulting in a cumulative frequency of themes exceeding the total number of studies included. Eleven recurring key themes were identified (Figure 2). Ethnic oral health inequalities (*n* = 35), access to dental care (*n* = 21), oral health in the Pacific Islands (*n* = 18), oral hygiene behaviours (*n* = 11), dental personnel (*n* = 6), oral cancer (*n* = 6), dental hospitalisations (*n* = 5), diet (*n* = 5), oral health promotion (*n* = 5), water fluoridation (*n* = 5) and dental anxiety (*n* = 3). The predominant themes reported were ethnic oral health disparities (36.8%), access to dental care (22.1%), and oral health diseases in the Pacific Islands (18.9%).

### 3.8 Inequities

Over time, studies have consistently reported poorer oral health outcomes among Pacific groups than among other ethnic groups. The most recent New Zealand studies reported Pacific ethnicity was associated with greater dental caries experience; in most studies, more Pacific children than other ethnic groups were categorised in the highest DMFT group (14, 15). Shackleton et al. (16) reported that the highest early childhood caries experience among 4-year-old children in New Zealand was among Pacific children (30.6%), whereas the lowest was among European children (6.8%). This is consistent with other evidence that Pacific children in New Zealand had fewer restorations but more extractions than other ethnic groups (17).

Among patients at New Zealand's University of Otago's dental faculty, Pacific patients were more likely to have one or more teeth extracted during a six-year period than patients of other ethnicities. The study also reported that Pacific patients were less likely to receive endodontic treatment, dental crowns, and in-office preventive services, such as smoking cessation advice and topical fluoride treatment (4).

As appropriate dental services are less available to Māori and Pacific children, Jamieson and Koopu (17) recommended examining whether the disparities are caused by acculturation or poverty. Oral health strategies should be tailored to meet the specific needs, backgrounds, and cultures of Pacific children in New Zealand to effectively promote oral health and help make culturally acceptable food choices and lifestyles.

A study of older New Zealanders (age 65+) found that proportionally more Pacific participants had retained their dentitions than participants of other ethnic groups (18). However, this finding appears to be an exception to the general trend of oral health inequities experienced by Pacific people across various age groups and settings. The study did not explain this finding, highlighting a need for further research to investigate the underlying factors contributing to better dental retention among older Pacific individuals.

### 3.9 Access to dental care

Many studies have highlighted barriers impeding Pacific peoples' access to dental care. Despite the provision of basic free oral health care for children under the age of 18 in New Zealand, the proportion of Pacific children accessing services remains disproportionately low. Poor dental attendance among Pacific children has been persistent in New Zealand. Paterson et al. (19) found that children from low-income households were less likely to receive dental care than children from high-income households. A New Zealand study also found that only 67.4% of Pacific students aged 13–17 years accessed dental health care in the prior 12 months, compared to 84.8% of European students (20). Pacific adolescents have also been found to visit private dental practices at significantly lower rates than adolescents of other ethnicities (21). Jamieson and Koopu (17) reported that Pacific children had the highest percentage of irregular dental attendance than other children of other ethnicities.

Enrolment percentages among Pacific and Māori children (83%) in New Zealand's Community Oral Health Services were also considerably lower than non-Māori and non-Pacific pre-schoolers (98%) (22). In New Zealand, a nationwide programme called “B4 School Check” offers free health and development checks for 4-year-olds; Gibb et al. (23) found that children of Pacific ethnicity were the least seen ethnic group.

A report from the New Zealand Health Quality & Safety Commission (22) urged in-depth evaluation and research to discover models of care best suited to Pacific children and their families. Māori and Pacific people should lead such research to ensure that planning and implementations are relevant to both groups (8, 24).

Cost is a predominant reason Māori and Pacific adults experience a greater severity of oral health diseases than other ethnic groups in New Zealand (24). More dated research found that Pacific people receiving a government benefit were more likely to use dental services than those in paid employment (7). Jatrana and Crampton (25) suggested the importance of a co-designed payment regime that assures the affordability of dental care. Socioeconomically disadvantaged individuals require additional help to take opportunities to improve oral health outcomes (26).

A longstanding issue among Pacific people appears to be that they access dental care when in pain. For example, a New Zealand study found that non-traumatic dental presentation attendances in the District Health Boards were by young Pacific people and Māori men seeking toothache or dental abscess relief (27). Previous research in Fiji reported that 63.2% of participants had not visited the dentist in the past 12 months, as they believed nothing was wrong (28). Pacific people have been described as episodic dental attenders, usually presenting because of pain (7).

Similarly, a small number of Pacific people on the Islands underwent routine dental examinations. For example, a study in the Solomon Islands found that only 5.3% of the participants had routine dental examinations, while more than half had never utilised dental services (53.3%) (29). In addition, only 8.3% of participants utilised dental services in the past year; the reasons included being in pain (74.4%), and treatment (14.5%) (29). Dated research in Fiji found that 89% of residents in institutions for older adults and people with disabilities could not recall their last dental examination (30). Paterson et al. (41) suggested that strategies specifically allowing Pacific people to reshape their understanding of oral health would reduce barriers to accessing dental healthcare.

One study found that Pacific adolescents valued dental aesthetics and function over oral and general health links. The participants stated that barriers to accessing dental care included negative perceptions of dentists, loss of structured support for attendance, unsupportive dental environments, and inconsistent guidance and oral care behaviours among parents and caregivers (31). Teevale et al. (20) also found that barriers to Pacific participants seeking primary health care included uncertainty about how to access primary health care, difficulties in contacting their healthcare providers, and a perceived lack of concern about oral health.

Pacific adolescents in New Zealand suggested that the accessibility to creating appointments, creating youth-friendly dental clinics, and offering rewards for attendance, may increase dental care attendance among Pacific adolescents. Further recommendations were to include younger Pacific and Māori oral health professionals and offer oral health promotion to parents and wider Pacific communities (8). Similarly, to increase the uptake of oral health services among Pacific adolescents in New Zealand, Kanagaratnam and Schluter (32) recommended encouraging more Māori and Pacific students to enter the dental professions.

### 3.10 Oral health conditions in the Pacific islands

Studies focusing on oral health in the Pacific Islands frequently reported the prevalence of dental caries and periodontal disease, with fewer studies on preventive measures.

Based on evidence from the current and older literature, the high prevalence of dental caries and periodontal disease has been a persistent issue in the PICTs. For example, in a study of children in Palau, 85% were found to have at least one decayed or filled tooth and dental caries (3). This study also found that dental caries was more prevalent among children in public schools than in private schools. In the Marshall Islands, at least 88% of children had at least one decayed tooth, and 65% experienced extensive caries (33). More dated research among Tuvaluans found calculus among 97% of 35–44 year-olds and caries among 92.5% of 6-year-old children (34). Among children in New Caledonia, European children had the lowest prevalence of dental caries (35).

A dated study in Fiji reported that those living in rural parts of the Pacific Islands experienced poorer oral health than those in urban areas. Older adults in the urban areas of Fiji were 53% less likely to report chewing difficulties than their rural counterparts (26). Fiji urban residents were also 31% less likely to report oral health issues than rural residents (26). However, a contrasting observation was made in a literature review on oral health in the PICTs, stating a higher DMFT prevalence in urban areas than in rural areas (36).

In 2010, Doherty et al. published a literature review on oral health in the Pacific Islands; the study established a lack of data and identified that oral health surveys were not conducted regularly in the Pacific Islands. In addition, the literature review stated that higher DMFT scores were found in urban areas of the Pacific Islands than in rural areas, and preventive measures, such as fissure sealants and fluoridation schemes, effectively reduced caries among the Pacific population (36).

### 3.11 Dental anxiety

Only two studies (1.8%) specifically examined dental anxiety among Pacific peoples. Prior research has associated dental anxiety with irregular dental visiting patterns (37). Among Pacific students enrolled at a University in New Zealand, dental anxiety was higher among females than males, and the overall prevalence of dental anxiety was low (18%) (38). An older study conducted in Fiji also found that Indigenous Fijians generally displayed low levels of dental anxiety without significant gender differences (39). However, Fijians of Indian descent reported decreased anxiety with older age and higher levels than indigenous (iTaukei) Fijians (39). Given that dental anxiety is a known barrier to dental treatment and the limited research on dental anxiety among Pacific peoples, more studies are required.

### 3.12 Oral hygiene

The review identified 12 (10.9%) studies that reported oral hygiene among Pacific people. The majority of the studies on oral hygiene focused on children's toothbrushing behaviours.

A New Zealand cohort study found that only 15% of Pacific 4-year-olds received adult assistance with toothbrushing (40). Another study from the same cohort highlighted a significant link between poor maternal oral hygiene behaviours and an increased risk of fillings and extractions in their children (41). Pacific and Māori children in New Zealand were less likely to brush their teeth than New Zealand European children (5, 6). Among New Zealand children, Pacific children had the lowest use of non-fluoridated pastes at 2.8% vs. 10.3% among Asian children (42).

Oral hygiene deficiencies exist in the Pacific Islands. The use of toothpaste among participants in the Solomon Islands was less than half (46.3%), with only 12.3% of adults brushing their teeth at least twice daily, as reported by Pengpid and Peltzer (29). In Fiji, just over a third (38%) of long-stay institutional residents did not practice oral hygiene care (30).

### 3.13 Dental hospitalisations

Only four (3.6%) studies investigated dental hospitalisation patterns in the Pacific population. In 2020, the potentially preventable hospitalisations for dental issues in New Zealand were over twice as high for Pacific children (1,397 per 100,000) compared to non-Pacific groups (554) (22). In the past, though, the opposite trend occurred, where Pacific children were less likely to receive hospital-based dental treatment under general anaesthesia than European children in New Zealand (5, 6). From 1996 to 1999, Pacific people had the second lowest rate of dental hospitalisations after Asians, and the highest dental hospital admissions were among New Zealand Europeans (44.4%) (43). More recently, annual non-traumatic dental presentations to government clinics have ranged from 66 to 103 per 10,000 Pacific people, surpassing 16–33 per 10,000 for non-Māori and other ethnicities (27).

### 3.14 Water fluoridation

Fluoridated water confers well-established caries prevention benefits. Pacific children in non-fluoridated communities experience higher caries rates than other children living in similar areas (44). In 2004, 42.7% of New Zealand 5-year-old Pacific children with a fluoridated water supply were caries-free, vs. only 32.5% in non-fluoridated areas (Ministry of Health (45). However, support for community water fluoridation remains mixed. In 2001, only 38% of Māori and Pacific adults favoured adding fluoride, while over half of Pacific respondents (59.4%) were unsure (43). An older study recommended expanded water fluoridation in the Pacific Islands to help address the high prevalence of caries (46). This highlights the ongoing challenge of integrating effective public health measures with the perceptions and preferences of the community.

### 3.15 Oral health promotion

Limited evidence exists on oral health promotion initiatives for Pacific groups. A program in Tonga named “ Mali Mali” successfully decreased the mean DMFT and increased toothbrushing frequency (47). In Fiji, most people reported learning oral health instructions from their dental personnel (31%) or medical doctors, relatives, friends, and media sources (28).

Earlier research has urged further research to determine effective strategies to motivate people through oral health education. In this context, chiefs in Samoa are recognised as respected and influential persons. This is important for oral health promotion purposes, as key oral health education can be effectively leveraged through chiefs and traditional structures to improve oral health outcomes (48). Community-based oral health promotion and prevention strategies should be considered to reduce the oral health burden across all generations, focusing on common risk factors and involving allied healthcare providers (40). Unless oral health promotion becomes more culturally appropriate and effective, it is unlikely that the dental health care system will reduce ethnic inequities (49).

### 3.16 Oral cancer

Pacific cancer data has historically been scarce (1). Only five (4.6%) studies reported oral cancer among Pacific people. Chelimo and Elwood (50) found the highest incidence of oropharyngeal cancer among Pacific groups in New Zealand despite having the lowest rates earlier. From 1999 to 2006, 24.2% of Auckland's Oral Medicine Clinic patients from the Pacific Island community had a confirmed histological diagnosis of oral lichen planus (51).

In older research from Papua New Guinea, a higher prevalence was reported for oral cancer than for stomach cancer and hepatoma, with an increasing incidence of oral cancer noted over time (52). Similarly, Moore et al. (1) found that the most common neoplasm in Papua New Guinea was oral cancer and identified combinations of tobacco smoking and betel quid chewing as primary risk factors. It has been noted that more males presented with cancers of the oral cavity and pharynx than females in Fiji, Guam, Papua New Guinea, Samoa, Solomon Islands, and Vanuatu (53).

### 3.17 Diet

The PICTs were previously known for having immaculate teeth (54). However, the effects of globalisation may have led to increased consumption of sugary foods and drinks, and tobacco, thus worsening oral health outcomes. Dental caries became more common following increased European trading and migration into the region (55). A dated analysis of New Zealand children's television programmes (which included 937 advertisements) found that 63% of the foods advertised were high in fat and sugar, whilst traditional staples, such as kumara, taro, and coconuts, were absent (56). Wilson et al. (56) also suggested the need for investigations exploring advertising policies and encouraged health authorities to collaborate with the food industry for advertising to promote positive nutritional education. Further research is also needed to investigate the association between socioeconomic status and diet (54).

Betel nut chewing remains a tradition spanning Southeast Asia to the Pacific but significantly elevates cancer risk (57). Only two (1.8%) sources reported betel nut chewing. Finau (58) reported that oral carcinoma associated with betel nut or betel lime chewing in Papua New Guinea affected 12% of people aged 35–44. Additionally, a study among Palauan people found that 77% of the sample reported chewing betel nuts with tobacco, and participants showed little concern about the impacts of betel nut chewing on their oral health (57). To promote behavioural changes, Quinn Griffin et al. (57) suggested involving nurses in developing health policies and ensuring clinical services are culturally sensitive to betel nut chewers.

### 3.18 Dental personnel shortages

The lack of dental personnel in the Pacific Islands was highlighted in 4% of the studies. Historically, there has been a considerable variation in the population-to-dentist ratio across the Pacific region (36). In 1976, the population per dentist ratio in the Western Pacific region was 1:4,533. At that time, it was recommended that the World Health Organization should plan training the dental health workforce in close conformity with the country's oral health needs (59).

By 2001, the Secretariat of the Pacific Community had deemed workforce shortages as a significant challenge, with little changing even decades later. A 2017 report documented only one dentist per 10,000 total population in the Pacific (47).

These shortfalls have significant intergenerational impacts. Because there is a lack of Pacific oral health providers, Pacific children in New Zealand are less familiar with dental environments, which poses a barrier to accessing oral health care. Pacific adolescents in New Zealand have suggested increasing the number of Pacific oral health professionals to enhance their dental attendance, as this would help address language and cultural barriers (8). Therefore, building workforce diversity is acknowledged as vital (60). Researchers have also urged the expansion of the Pacific dental workforce (20).

## 4 Discussion

This scoping review was conducted to map and synthesise the existing knowledge base on Pacific oral health, revealing significant knowledge gaps in targeted oral health strategies and solutions. The amount of Pacific oral health research published has been increasing since 2000. However, although most Pacific Island countries have routine health data collection systems, there are significant gaps in publication, dissemination, and utilisation to inform health policy and decision-making (61).

Although the studies included in this review vary in terms of geographic focus, it is important to note that the majority of studies and first-named authors were based in New Zealand. This reflects on the sizable Pacific diaspora populations residing there. However, the social and economic conditions faced by Pacific populations in New Zealand may differ from those in different countries which creates a challenge in generalising findings for Pacific people around the globe. Variations in cultural practices, health care systems and social conditions in different countries contribute to differing oral health experiences. This heterogeneity underscores the need of more studies to be conducted in other major countries where Pacific diaspora groups reside, such as Australia and the United States, to understand the oral health of Pacific people globally.

A relatively small fraction of studies has focused exclusively on Pacific populations. Most involved comparisons to other ethnic groups, highlighting the persistent oral health inequities faced by Pacific peoples. These disparities likely stem from complex interactions of social, economic, and environmental determinants, negatively impacting socioeconomically disadvantaged Pacific families (80). There is a need for more holistic research approaches encompassing the broad determinants of oral health among Pacific peoples. Despite longstanding concerns, this review revealed major gaps in oral health promotion initiatives tailored to Pacific cultural contexts. Investigating Pacific peoples' perspectives could provide vital insights to guide such initiatives. Additionally, understanding the effects of culture on oral health may help inform the development of effective policies to reduce oral health inequalities among Pacific people.

This study has highlighted that Pacific people experience multiple barriers to oral health care, which is a significant issue affecting their oral health. This study identified several important themes, such as ethnic oral health inequalities, access to dental care, oral conditions, dental anxiety, oral hygiene behaviours, dental hospitalisations, water fluoridation, oral health promotion, oral cancer, diet, and the lack of dental professionals which can directly affect oral health in the Pacific. A greater understanding of these specific issues can lead to better-targeted interventions and healthcare strategies.

Inequalities in health and health-related outcomes based on race and ethnicity exist within and between countries (62–64). This review found that Pacific people have had higher caries experiences and dental extractions. The impact of ethnic oral health inequalities, especially in children, has been prominently highlighted in other studies related to oral health behaviours (52, 65–68). While many studies did not report on any ethnic differences in terms of the age at which children start brushing or first dental attendance (66, 69), a Canadian study reported marked ethnic inequalities in regular adult tooth brushing (52). This parallels the findings of this study, where the perceived and clinical oral health status of adults from minority ethnic groups was poor (70–72).

The World Dental Federation (73) highlighted some key barriers to the accessibility and utilisation of oral healthcare. These can be further grouped into socioeconomic factors, which present substantial challenges to individuals seeking oral health care (including the cost of dental care, education levels, income, and unavailability of health insurance), human resources (lack of trained personnel and uneven distribution of workforce), and health behaviours and beliefs (including lack of preventive care and lifestyle that is not conducive to oral health). Across different studies, a common theme related to accessibility is seen, which compromises the oral health of Pacific people, as found in this study.

Most of the literature on oral health conditions and hospitalisation among the Pacific people in this study is not recent, as very little research specifically focuses on oral health in PICTs (54). This could be due to a lack of published data on this subject or research conducted in the region (61). Furthermore, similar to the findings of this study, dental caries and periodontal disease have been identified as the most common oral health conditions affecting PICTs (54). Before the effects of globalisation, Pacific people had good oral health; however, an older study stated that the increased consumption of refined sugar and tobacco has led to an increase in dental caries in children and periodontal disease in adults (55). Community-based preventive initiatives that target key risk factors such as refined sugar consumption and tobacco may be effective in addressing these behaviours.

The data on oral cancer in this study can not represent the actual situation in the Pacific, as few studies on this from the region have been published. Ng et al. (74) reported that the lack of national cancer control strategies and registries and the limited availability of trained personnel add to the burden of oral cancer in the Asia-Pacific region. Papua New Guinea has been reported to have the highest rate of oral and oropharyngeal cancer in the world. By 2030, the incidence in Oceania will increase by 45% compared to that in 2012 (75). Tobacco, alcohol, and areca nut (betel quid) use are the main factors contributing to oral and oropharyngeal cancer (74). Moreover, chewing of areca nut has been ranked as the fourth most common form of substance abuse after nicotine, ethanol, and caffeine (76, 77). Awareness regarding cessation of areca nut chewing can be challenging in the Pacific, as it is part of the culture and identity of the Melanesian community in the PICTs (78, 79).

To our knowledge, this is the first formal scoping review, collating the findings of a diverse range of studies on Pacific oral health over nearly a 70-year period. To ensure that all information on Pacific oral health was included in the study, it was limited by not assessing the quality of sources of information. The exclusive use of English-language searches may have overlooked relevant studies published in other languages, particularly French, given the presence of French territories in the Pacific. While grey literature was included, some government reports or local publications may still have been missed due to search limitations. The absence of a publicly available protocol, which is becoming more common for scoping reviews, is a study limitation. Furthermore, the review is restricted to studies published up to 2023. Another potential limitation is that many studies did not specify subpopulations, which may not provide information about specific cultural or socio-economic factors impacting oral health in different Pacific communities. As the majority of the studies were concentrated in New Zealand, this geographical skew may limit the generalisability of the findings to other Pacific regions.

Oral health research was conducted in partnership with Pacific communities to translate evidence into practice and policy changes that can improve oral health outcomes. There is a need for longitudinal studies in the Pacific that track oral health outcomes over time, as well as comprehensive research that integrates various aspects of oral health, from clinical conditions to preventive care and health promotion.

## 5 Conclusion

Pacific people tend to experience poorer oral health than people of other ethnic groups. Further research is needed to investigate Pacific peoples' perspectives and experiences on oral health and access to dental care, and to inform the development of culturally-appropriate, effective strategies to address these issues. Pacific peoples' insights are vital in developing health promotion initiatives and recommendations, which Pacific professionals and policymakers should ensure cultural relevance and efficacy. Additionally, expanding the Pacific dental workforce and research capabilities is essential for increasing the uptake of oral health services, developing interventions that can meet the needs of Pacific communities, and fostering oral health equity.
